# Kaminari: a frugal colored index for approximate *k*-mer queries

**DOI:** 10.1093/bioadv/vbag120

**Published:** 2026-04-26

**Authors:** Victor Levallois, Yoshihiro Shibuya, Bertrand Le Gal, Yoann Dufresne, Rob Patro, Pierre Peterlongo, Giulio Ermanno Pibiri

**Affiliations:** GenScale, University of Rennes, Inria, CNRS, IRISA—UMR 6074, Rennes, F-35000, France; Sequence Bioinformatics Unit, Institut Pasteur, Paris, F-75015, France; University of Rennes, Rennes, F-35000, France; Sequence Bioinformatics Unit, Institut Pasteur, Paris, F-75015, France; Univ. Lille, CNRS, Centrale Lille, UMR 9189 CRIStAL, Lille, F-59000, France; Bioinformatics and Biostatistics Hub, Institut Pasteur, Université de Paris, Paris, F-75015, France; Department of Computer Science, University of Maryland, College Park, MD 20440, United States; GenScale, University of Rennes, Inria, CNRS, IRISA—UMR 6074, Rennes, F-35000, France; DAIS, Ca’ Foscari University of Venice, Mestre, I-30172, Italy; ISTI-CNR, Pisa, I-56124, Italy

## Abstract

**Motivation:**

Identifying which documents in a large database contain a query string is a fundamental problem in Information Retrieval and Computational Biology. We focus on the approximate version of this problem for genomic sequences: the result set may contain false positive matches but no false negatives. State-of-the-art solutions rely on Bloom filters to index all *k*-mers (substrings of fixed length *k*) in the documents. To answer a query, documents sharing at least a user-prescribed fraction of query *k*-mers (typically 75%–80%) are returned.

**Results:**

Here, we explore an alternative index design based on *k*-mer minimizers and integer compression methods. We show that a careful implementation of this design outperforms previous solutions based on Bloom filters by a wide margin: the index has lower memory footprint and faster query times, while false positive matches have only a minor impact on the ranking of the documents reported. This trend is robust across genomic datasets of different complexity and query workloads.

**Availability and implementation:**

The software is freely available at github.com/yhhshb/kaminari under the MIT license. Reproducibility scripts are available at github.com/vicLeva/benchmarks_kaminari.

## 1 Introduction

Let $R=\{R_{1},\ldots ,R_{N}\}$ be a collection of textual documents. Efficiently identifying which documents in R contain a query string *Q* is a fundamental problem, especially in fields like Information Retrieval and Computational Biology. This work focuses on a specialized case where the documents are DNA strings, using the alphabet Σ={A,C,G,T}. For large-scale processing, it has become customary to represent a DNA string by its collection of *k*-long substrings (named “*k*-mers”). To assess whether *Q* is present in a document $R_{i}$, we compute the number of *k*-mers of *Q* that are also substrings of $R_{i}$. If this number is at least 75%–80% of the total *k*-mers in *Q*, we consider *Q* to appear in $R_{i}$ (Ukkonen 1992). Therefore, $R_{i}$ can be viewed as a (multi-)set of $|R_{i}|-k+1$  *k*-mers. However, *k*-mers of *Q* may appear in different order in $R_{i}$, which would mean *Q* does not actually appear as a substring. This situation is unlikely unless *k* is very small. In this work we study efficient solutions to the following problems.

Problem 1 (Exact colored *k*-mer indexing.) *Build a data structure, referred to as the index in the following, that allows to retrieve the set* $C_{k}(x)=\{i|x\in R_{i}\}$  *as efficiently as possible for any k-mer* $x\in \Sigma^{k}$*. If x does not occur in any* $R_{i}$*, then* $C_{k}(x)=Ø$.

In other words, the set $C_{k}(x)$ contains all the identifiers, called “colors,” of the documents where the *k*-mer *x* appears. We refer to $C_{k}(x)$ as the *color set* of *x*.

While exact solutions to Problem 1 have been studied extensively (Karasikov *et al.* 2020, 2022, Alanko et al. 2023, Fan *et al.* 2023, 2024), *approximate* solutions—allowing for potential *false positive* matches but no *false negatives*—are gaining importance for their potential enhanced performance and scalability. Although an acceptable percentage of false positive matches can be dealt with during downstream analyses, we do not consider solutions that generate false negative matches and potentially miss out on fundamental data sets at query time. In this work, we thus consider the approximate version of this problem, defined as follows.

Problem 2 (Approximate colored *k*-mer indexing.) *Build an index allowing retrieval of set* ${\tilde{C}}_{k}(x)⊇C_{k}(x)$  *as efficiently as possible, for any k-mer* $x\in \Sigma^{k}$  *and with small* $\left|{\tilde{C}}_{k}(x)\setminus C_{k}(x)\right|$.

The primary motivation for studying solutions to Problem 2 is to create a more efficient method for computing ${\tilde{C}}_{k}(x)$, requiring less RAM and CPU time than $C_{k}(x)$. As a drawback, ${\tilde{C}}_{k}(x)$ is a superset of $C_{k}(x)$, which may lead to false positives (elements in ${\tilde{C}}_{k}(x)\setminus C_{k}(x)$) that do not actually contain the *k*-mer *x*.

State-of-the-art solutions to Problem 2 (reviewed in Section 3) are primarily based on *Bloom filters* (Bloom 1970)—the most well-known approximate membership data structure. In these solutions, a Bloom filter is created for each document by inserting all its *k*-mers in the filter. The set of filters is then laid out as a matrix (Bingmann *et al.* 2019, Bradley *et al.* 2019, Lemane *et al.* 2022, 2024), or in a tree hierarchy (Sun *et al.* 2018, Harris and Medvedev, 2020, Mehringer *et al.* 2022, Marchet and Limasset 2023). As argued below, these approaches are space-inefficient and do not exploit some important properties of *k*-mers to obtain better performance for Problem 2.

**Our contribution.** We contribute an alternative index design that does not use Bloom filters but is based on *k*-mer minimizers (Schleimer *et al.* 2003, Roberts *et al.* 2004) and integer compression techniques (Pibiri and Venturini 2021) to address the main limitations of the state of the art.

In short, our solution merges the color sets of *k*-mers in a coherent way, e.g. those that share the same minimizer (smallest substring). This allows to save storage space (as the number of indexed sets reduces dramatically). Unlike Bloom-filter based solutions, where approximate color sets are created by merging the color sets of *k*-mers drawn at random (*k*-mers that hash to the same bit positions in the filter), we merge them based on their “similarity.”

While this merging strategy still allows false positives, they minimally affect the result sets computed by our index in the following sense. For a query *Q*, each reported document *D* in the result set is associated with a *score*, being the number of *k*-mers of *Q* that *D* contains. Retrieved documents are sorted by decreasing score, allowing for ranking results. We argue that most false positives do not have a score able to *significantly alter the ranking of the result set* compared to an exact solution. To assess this result, we use the established similarity measure called *rank-biased overlap* (Webber *et al.* 2010).

These methods have been implemented in a software called “Kaminari” (雷, “thunder” in Japanese), freely available on GitHub.

We conducted an extensive experimental analysis, comparing Kaminari with other efficient approximate and exact indexes. Results show that Kaminari produces smaller indexes and faster queries than other approaches. Kaminari is also competitive in terms of time and resources used to build the index. Lastly, we demonstrate that false positives have a small impact on the user, as the most relevant results of a query remain trustworthy.

## 2 Background

### 2.1 Sampling and hashing

Definition 1 (Minimizer sampling.) *A minimizer sampling scheme is defined by a triple* (m,k,O)*, with* m,k∈N, m<k*, and* $O:\Sigma^{m}\to R$  *is an order over all m-long strings. Given a k-mer x, the minimizer* μ  *of x is the leftmost m-mer of x such that* O(μ)≤O(y)  *for any other m-mer y of x.*

In practice, O is usually implemented as a non-cryptographic pseudo-random hash function, such as MurmurHash2 (Appleby 2016), obtaining the so-called “random” minimizer scheme (Schleimer *et al.* 2003, Roberts *et al.* 2004). (We however use the simple lexicographic order for ease of visualization when discussing the examples.) To simplify notation, “Minimizer (x)” designates the minimizer of the *k*-mer *x*, without specifying parameters *m* and O. Also, Minimizer (Q) is the (multi-)set of all the minimizers of the *k*-mers of the string *Q*.

A string is said *sampled* at the positions of the minimizers of its *k*-mers. For a string composed of *n* i.i.d. random characters and when *m* is sufficiently long, the expected number of distinct sampled positions is ≈2/(k−m+2)·(n−m+1) [see Theorem 3 by Zheng *et al.* (2020)] for details and the article by Koerkamp and Pibiri (2024) for a recent overview of sampling algorithms).

Definition 2 (Minimal perfect hash function (MPHF).) *Let* f:U→{1,…,n}  *for some universe set U. The function f is said to be a minimal perfect hash function for the set* S⊆U*, with* |S|=n*, if* f(x)≠f(y)  *for all* x,y∈S, x≠y.

In simpler words, a MPHF for the set *S* maps its *n* keys bijectively into the first *n* natural numbers. The theoretical space lower bound (Mehlhorn 1982, Mairson 1983) for representing a MPHF is $n{\;\text{log}\;}_{2}e-O(\text{log}\;n)$ bits assuming |U|→∞, which is approximately 1.443 bits per key for large *n*. Practical constructions with just 0.1% overhead on top of the lower bound have been proposed (Lehmann *et al.* 2025). In this work, we use the PTHash data structure (Pibiri and Trani 2024, Hermann *et al.* 2024), optimized for fast queries with a space usage of 2−3 bits/key.

### 2.2 Query mode

Given a multi-set *M*, we indicate with w(i,M) the multiplicity of element *i* in *M*. We colloquially refer to w(i,M) as the *score* of *i* in *M*. For clarity, we recall that A⊎B denotes the multiset union, *e.g.* {a,a,b}⊎{a,b,c}={a,a,a,b,b,c}.

Definition 3 (Threshold-union query.) *Let Q be a query string with* |Q|≥k  *and* $M(Q)={⊎}_{x\in Q}C_{k}(x)$  *be the multi-set union of the color sets for all the k-mers of Q. For a given* 0<τ≤1*, the threshold-union query computes the list* R(Q,τ)  *that is the set* {i|w(i,M(Q))≥⌊τ(|Q|−k+1)⌋}  *where the colors i are sorted by decreasing score* w(i,M(Q)).

Note that this definition does not consider the abundance of each *k*-mer x∈Q in the document identified by the color *i*.

**Figure 1:**
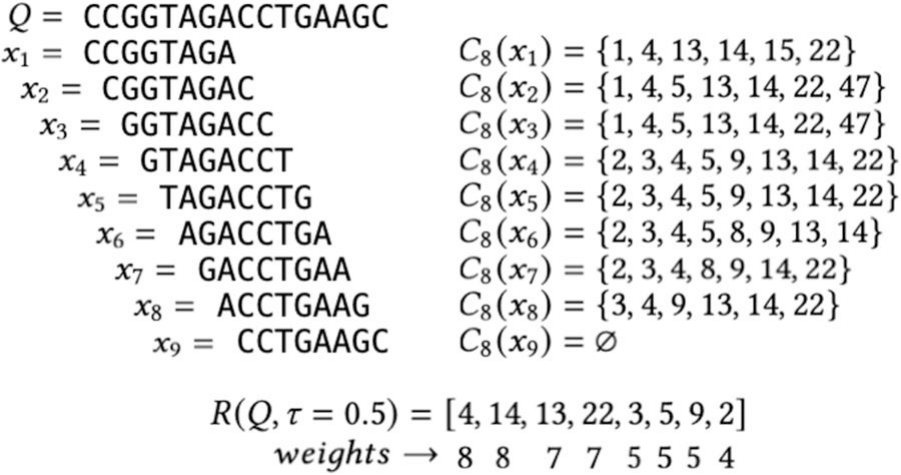
Threshold-union query example for k=8 and τ=0.5. The query string *Q* contains 9 *k*-mers, $x_{1},\ldots ,x_{9}$. On the right, their hypothetical color sets. The fact that $C_{8}(x_{9})=Ø$ means that $x_{9}$ has not been indexed. The result set R(Q,τ) is computed by including all colors whose weight is at least ⌊τ(|Q|−k+1)⌋=⌊0.5·9⌋=4, sorted by weight.

We say that R(Q,τ) is a *ranked list*, as each color is ranked by its score. A common value for τ is, for example, 0.8, retaining documents containing at least 80% of the *k*-mers of *Q*. This query mode is used by both exact and approximate indexes in state-of-the-art methods [e.g. MetaGraph (Karasikov *et al.* 2020), Fulgor (Fan *et al.* 2024), COBS (Bingmann *et al.* 2019), and kmindex (Lemane *et al.* 2024)]. Figure 1 shows an example of a threshold-union query. The threshold-union query algorithm is not meant to accelerate the query. It is not used for stopping computations when we detect that the threshold is reached or when we detect that it will not be reached.

The approximate version of R(Q,τ) is indicated with $\tilde{R}(Q,\tau )$ and is defined in an analogous way but over $\tilde{M}(Q)={⊎}_{x\in Q}{\tilde{C}}_{k}(x)$. Note that, since ${\tilde{C}}_{k}(x)⊇C_{k}(x)$ for any *x* we have that: (i) $\tilde{R}(Q,\tau )⊇R(Q,\tau )$ for any query *Q*, and (ii) $w(i,\tilde{M}(Q))\geq w(i,M(Q))$ for any color *i*.

### 2.3 Rank-biased overlap (RBO)

Given two ranked lists of infinite length, Webber *et al.* (2010) define their *rank-biased overlap* (RBO, henceforth) as a measure of their similarity.

In this work, we exploit one particular definition: the “bounded RBO,” noted RBO@D, that measures the RBO of lists truncated at depths *D*. See the Appendix, available as supplementary data at *Bioinformatics Advances* online, for details and examples.

## 3 Related work

Since this work focuses on approximate solutions, we do not review exact indexes here. However, our experiments also report results for two exact indexes, MetaGraph (Karasikov *et al.* 2020) and Fulgor (Fan *et al.* 2024), used to determine the ground truth for the queries.

Most solutions use approximate membership query data structures, mainly indexing *k*-mers with Bloom filters (Bloom 1970). Practical implementations include BIGSI (Bradley *et al.* 2019), later enhanced by COBS (Bingmann *et al.* 2019). These methods create individual Bloom filters for each document, forming final indexes as inverted matrices that interleave the filters. For a given hash value, *N* consecutive bits indicate the presence/absence of a *k*-mer across *N* documents, allowing efficient query access to all *k*-mer occurrences per sample.

Recent advancements in MetaProFi (Srikakulam *et al.* 2023) and kmtricks (Lemane *et al.* 2022) have enabled the processing of larger data volumes. Notably, kmtricks was utilized in kmindex (Lemane *et al.* 2024) to index hundreds of terabytes of metagenomic seawater data for the first time. This method employs the findere approach (Robidou and Peterlongo 2021), where a unique hash function is used to reduce false positive rates by querying multiple *s*-mers per *k*-mer (with s≤k). This approach lowers query times by minimizing accesses to the bloom filter. Similarly, MetaProFi utilizes a chunked Bloom filter matrix with compression to significantly decrease the overall index size.

A family of methods organizes Bloom filters in a tree topology (Solomon and Kingsford 2016, Sun *et al.* 2018, Harris and Medvedev 2020, Gupta *et al.* 2021, Marchet and Limasset 2023), with leaves containing Bloom filters and internal nodes storing unions of siblings. Such layout saves space by avoiding duplicating information related to *k*-mers present in a full subtree and enhances query speed by halting searches as soon as a subtree is fully determined. Nevertheless, these approaches suffer from random memory access issues that limit their query performances.

Finally, the tools Raptor (Seiler *et al.* 2021) and PebbleScout (Shiryev and Agarwala 2024) are the closest solutions to our proposal. They index color sets of minimizers of *k*-mers rather than the *k*-mers themselves. For Raptor, minimizers are indexed with Hierarchical Interleaved Bloom Filters (Mehringer *et al.* 2022), optimizing for unbalanced input dataset sizes. In PebbleScout, color sets are indexed using minimizers of fixed length m=25, and *k*-mers of length k=42. While PebbleScout has been effectively used on a significant portion of the Sequence Read Archive (SRA) datasets (Katz *et al.* 2022), it is not open-source, preventing a direct comparison. Lastly, minimizers have also been exploited to map *k*-mers to reads (Vandamme *et al.* 2025).

## 4 Approximate indexing of a set of documents

The high-level idea we propose is to store color sets of minimizers, instead of color sets of *k*-mers. In other words, we take the union of color sets of *k*-mers sharing the same minimizer. For the sake of clarity, we define $C_{m}(\mu )$ as the color set of a minimizer μ, i.e. $C_{m}(\mu )=\{i|\mu \in R_{i}\}$. In practice, our solution is to let ${\tilde{C}}_{k}(x)=C_{m}(M\text{INIMIZER}(x))$.

Minimizers have some important properties that should be exploited to achieve compact space and fast query time for Problem 2.

1. *Consecutive k-mers tend to share the same minimizer.* This property allows for a drastic reduction in the size of the final index since there are fewer distinct minimizers than *k*-mers. As reviewed in Section 2.1, we expect to have approximately (k−m+2)/2× fewer (random) minimizers than *k*-mers. Additionally, we exploit this property when retrieving the color sets of *k*-mers: we save repeated accesses to the color set of the minimizer (i.e. we cache the set) by *streaming* through the *k*-mers of *Q*. Other solutions, like COBS, cannot exploit this streaming query pattern because every *k*-mer lookup accesses a different row of its binary matrix, resulting in a cache miss per each *k*-mer of *Q*. Also, we *skip* the query of all *k*-mers whose minimizer is not indexed.
2. *Minimizers of sufficient length (e.g.* m=19  *for* k=31*) are highly specific and thus sparsely distributed across the document collection.* Consequently, we expect to associate with each minimizer a short color set, whose compressed representation typically occupies significantly fewer than *N* bits. This stands in contrast to solutions based on uncompressed binary matrices, such as COBS, which must always retrieve and decode a fixed-size vector of *N* bits regardless of the minimizer/*k*-mer’s specificity.
3. *Consecutive k-mers tend to have very similar color sets (if not exactly the same).* As noted above, consecutive *k*-mers tend to share the same minimizer. Because of this, there is a high chance that every time we see a minimizer we also see its *k*-mers. This intuitively helps to keep under control the amount of false positives in ${\tilde{C}}_{k}(x)$ for all the *k*-mers *x* that have minimizer μ because $C_{m}(\mu )$ results from the union of similar sets. On the other hand, several indexes reviewed in the previous section merge color sets of randomly-chosen *k*-mers potentially having very different sets of colors.

Some properties have been used in existing literature (Seiler *et al.* 2021, Pibiri 2022, Fan *et al.* 2024, Vandamme *et al.* 2025) to address related problems; however, before this work no comprehensive solution to Problem 2 integrating all these properties was available. Notably, these properties are independent of the specific data structures used for indexing minimizers and compressing color sets, allowing for various space/time trade-offs. We detail our approach in the following sections, introducing an index named “Kaminari” that leverages these properties.

### 4.1 Index creation and description

**Figure 2:**
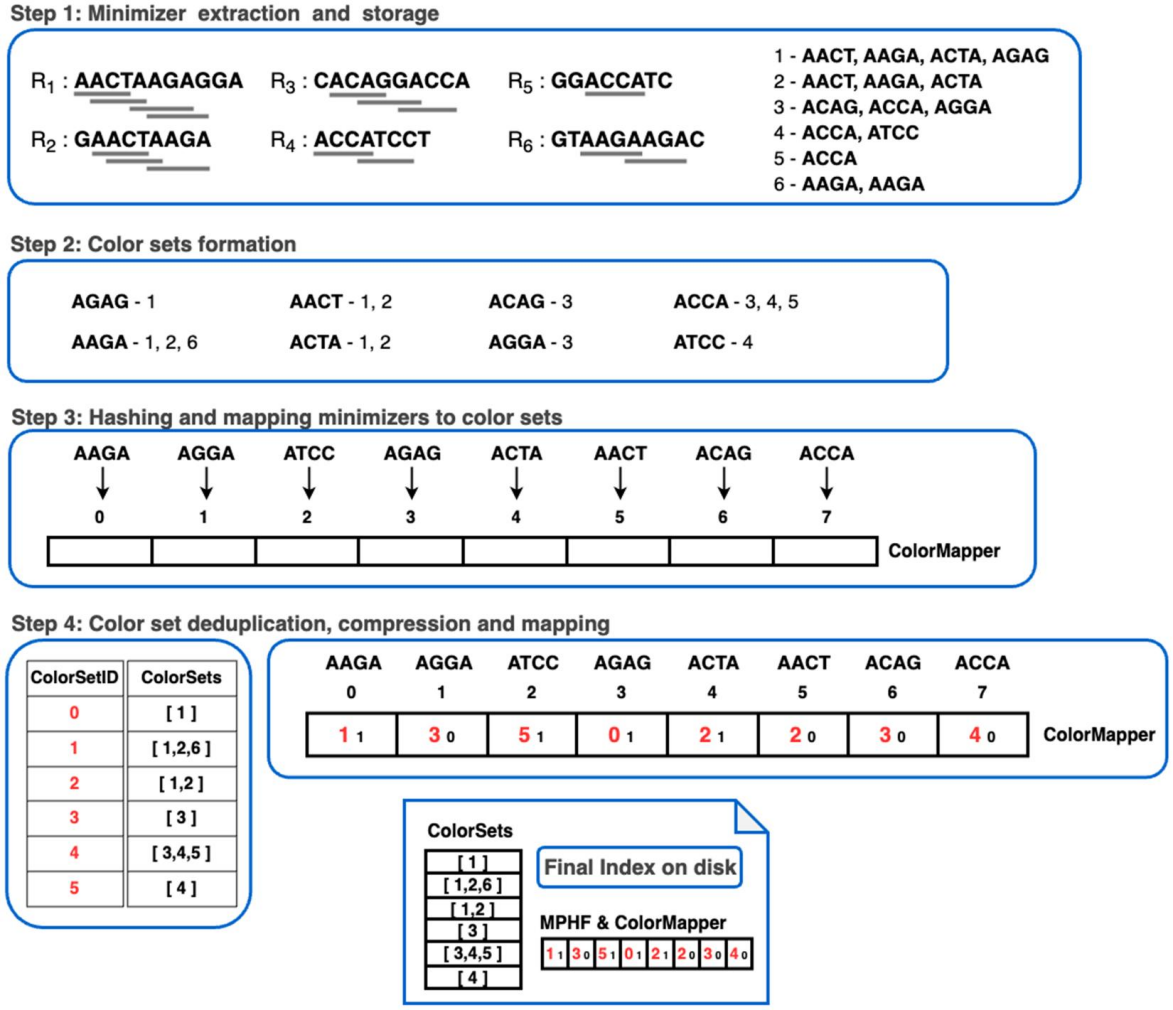
Index construction steps. The example uses k=6 and m=4. The *ColorMapper* table is populated at the end of Step 4, after deduplicating color sets.

The creation of a Kaminari index comprises four steps, depicted in Fig. 2.

**Step 1: Minimizer extraction and storage.** For each document $R_{i}\in R$, all random minimizers of length *m* are computed, and their MurmurHash2 hashes (assuming |Σ|=4 and m≤32 as in our experiments, we use 64-bit hashes) are stored in an external-memory vector. This vector maintains a list of disk files (blocks) containing its elements, along with a small internal-memory buffer for the block under construction. If the total RAM used by the *N* buffers exceeds a user-defined limit, the buffers are sorted, deduplicated, and written to *N* separate blocks on disk. Let K≥N be the total number of blocks written.

**Step 2: Color sets formation.** We merge these *K* sorted blocks to deduplicate minimizers and construct the corresponding color sets, utilizing a classic merging strategy with ${\;\text{log}\;}_{2}K$ parallel merges. This process also enables the incremental building of minimizer color sets, leveraging external-memory abstractions indicating each block’s logical color. Color sets can be encoded as binary vectors of *N* bits or through more advanced encodings (see below). Ultimately, we compute the distinct minimizers of R and their associated color sets.

**Step 3: Hashing and mapping minimizers to color sets.** Call *S* the set of distinct minimizers of R. A minimal perfect hash function (MPHF) *f* is built for *S* using PTHash (Pibiri and Trani 2021, 2024, Hermann *et al.* 2024). This function maps each minimizer to an index in a table, ColorMapper[1..|S|], created and used in the last step.

**Step 4: Color set deduplication, compression, and mapping.** Let *z* indicate the number of distinct color sets. Note that z≤|S| because different minimizers can have the same color set. We start by deduplicating color sets and assign a unique identifier 0<I≤z to each distinct color set (for example, following their lexicographic order). For every μ∈S, we store the pair of integers (I,F) at position i=f(μ) in the *ColorMapper* table:

1. The integer *I* uniquely identifies the color set associated with μ.
2. The value *F* is *b*-bit integer, computed as F=FINGERPRINT (μ). We implement the function $F\text{INGERPRINT}\;\Sigma^{m}\to [2^{b}]$ as a pseudo-random hash function, that is, *F* is a pseudo-random integer in the interval $\left[1,2^{b}\right]$. Such fingerprint is used in the detection of alien minimizers (minimizers not belonging to the input collection R). Suppose we query for a minimizer α. At query time, FINGERPRINT (α) is computed and compared against that stored in ColorMapper[f(α)]. If they are *not* the same, then α is surely alien. Otherwise, α is not alien with probability at least $1-1/2^{b}$. This is a folklore technique to implement a space-efficient static filter with prescribed false positive probability (Broder and Mitzenmacher 2003, Bender *et al.* 2018, Marchet *et al.* 2020). In the example of Fig. 2, we use b=1 and these bits are displayed in small black font within the *ColorMapper* table.

It follows that the *ColorMapper* table is stored in $\left|S|(b+\left\lceil {\;\text{log}\;}_{2}z\right\rceil \right)$ bits. The color sets themselves are compressed using techniques inspired by Fulgor (Fan *et al.* 2024). Specifically, each color set is classified into one of the following three categories based on its size.

- *Sparse color sets.* If the number of colors in the set is less than N/4, the colors are encoded using a difference-based approach: we first compute the differences between consecutive integers and then apply Elias’ δ encoding (Elias 1975) to compress them.
- *Dense color sets.* When the size of the set is at least N/4 and at most 3N/4, we use a binary vector of *N* bits. A bit set at position *i* indicates that color *i* is present in the color set.
- *Very dense color sets.* Lastly, if the size of the set exceeds 3N/4, we encode only the absent document identifiers using the same difference-based approach as for the sparse sets.

All the compressed representations of the color sets are concatenated in a single bitvector called *ColorSets* in Fig. 2. The starting positions of the color sets are kept in a separate list, so that we can access the compressed representation of the *i*th color set for any 0<i≤z. Since this list is monotone by construction, we compress it using the Elias-Fano encoding (Fano 1971, Elias 1974).

To sum up, the Kaminari index consists of the following three components: (1) the MPHF *f*, (2) the *ColorMapper* table, (3) and the compressed color sets.

### 4.2 Queries

The crux of computing $\tilde{R}(Q,\tau )$ is how $w(i,\tilde{M}(Q))$ is determined efficiently *without* explicitly materializing the set $\tilde{M}(Q)$, i.e. without taking the multi-set union of all the colors sets for all the *k*-mers of *Q*. As it is clear, we need to only consider the color sets for the minimizers of the *k*-mers of *Q*, Z=MINIMIZER (Q). Since each μ∈Z appears in *Q* for w(μ,Z) times by definition, the score of the color *i* in $\tilde{M}(Q)$ is the sum of the scores w(μ,Z) for all color sets $C_{m}(\mu )$ where *i* appears. In formal terms, $w(i,\tilde{M}(Q))=\sum_{\mu \in Z}w(\mu ,Z)\cdot I[i\in C_{m}(\mu )]$, where I[E] is the indicator variable for the event *E* (i.e. I[E]=1 if *E* is true and 0 otherwise). This allows us to efficiently compute $w(i,\tilde{M}(Q))$ in an incremental way (Algorithm 1): we initially set $w(i,\tilde{M}(Q))=0$ and, when scanning $C_{m}(\mu )$, sum w(μ,Z) to $w(i,\tilde{M}(Q))$ if $i\in C_{m}(\mu )$.

**Algorithm 1** The threshold-union query for a sequence *Q* and parameter τ, as supported by Kaminari. The algorithm computes the ranked list $\tilde{R}(Q,\tau )$ as described in Section 2.2, i.e. by returning all colors *i* such that $w(i,\tilde{M}(Q))\geq \tau (|Q|-k+1)$. For ease of notation, we let $w(i):=w(i,\tilde{M}(Q))$ and $\tilde{R}:=\tilde{R}(Q,\tau )$ in the pseudocode.
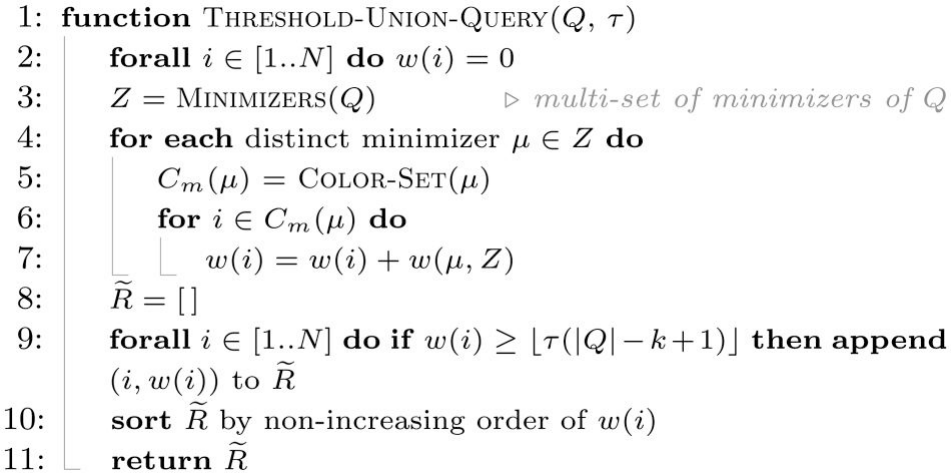

It remains to explain how the set $C_{m}(\mu )$ is retrieved from the index (Algorithm 2). First, p=f(μ) is computed and the pair (j,F)=ColorMapper[p] is retrieved. If *F* matches the fingerprint of μ, then we have $C_{m}(\mu )=\mathrm{ColorSets}[j]$; otherwise $C_{m}(\mu )=Ø$.

**Algorithm 2** The retrieval of the color set of the minimizer μ from a Kaminari index.
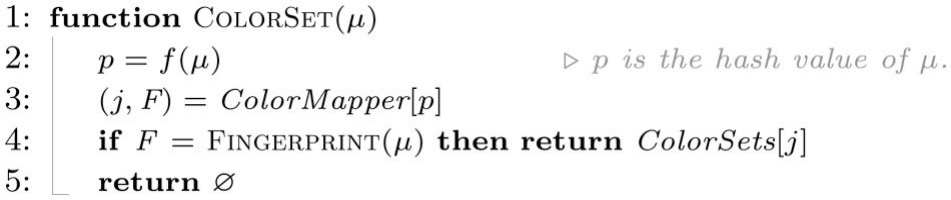

### 4.3 False positives

With the proposed scheme, false positives arise when computing ${\tilde{C}}_{k}(x)$ as $C_{m}(M\text{INIMIZER}\;(x))$ due to two distinct causes:

1. The first cause is that the color set of a *k*-mer is a subset of the color set of its minimizer, i.e. $C_{k}(x)\subseteq C_{m}(M\text{INIMIZER}\;(x))$. Hence using $C_{m}(M\text{INIMIZER}\;(x))$ as an approximation for $C_{k}(x)$ clearly introduces false positives, i.e. spurious colors that are due to $C_{k}(y)\subset C_{m}(M\text{INIMIZER}\;(y))$ for any other *k*-mer y≠x such that MINIMIZER (y)=MINIMIZER (x). This effect is exacerbated when the *k*-mer is absent but the minimizer is present. In this case, *all* the colors in the returned color set are false positives.
2. The second cause is that the MPHF *f*—by definition—cannot detect whether a minimizer has been indexed or not. For an alien minimizer μ, we remark that f(μ) can be any integer in {1,…,|S|} (where *S* is the set of distinct minimizers in the input). This implies that Kaminari always returns a color set, even when an alien minimizer is queried. In such cases, the correct color set would be the empty set and therefore all elements of the returned set must be considered false positives. To mitigate this effect, we use the *b*-bit fingerprint *F* which is stored along with the identifier of the color set of the minimizer. Figure 2 shows an example with b=1, so that we reject alien minimizers for approximately 50% of the time.

**Figure 3:**
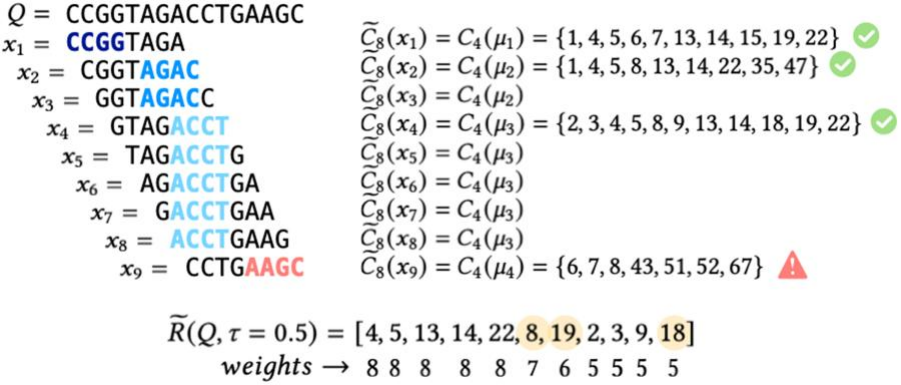
The same example discussed in Figure 1 but in the approximate setting presented in Section 4, i.e. assuming that ${\tilde{C}}_{k}(x_{i})=C_{m}(\mu_{j})$ for a *k*-mer $x_{i}$ whose minimizer is $\mu_{j}$. The lexicographic minimizer of length m=4 of each *k*-mer is indicated in bold font; we have $\mu_{1}=\mathrm{CCGG}$, $\mu_{2}=\mathrm{AGAC}$, $\mu_{3}=\mathrm{ACCT}$, and $\mu_{4}=\mathrm{AAGC}$. We assume that $\mu_{1-3}$ match the fingerprint stored in the index (check mark), whereas $\mu_{4}$ does not (warning symbol) and it is correctly labeled as alien. Compared to the exact result set R(Q,τ)=[4,14,13,22,3,5,9,2] from Figure 1, the set $\tilde{R}(Q,\tau )$ contains three false positives, {8,19,18} (indicated by the shaded circles), and the ranking of the reported colors is different.

Figure 3 illustrates an example of these two effects, using the same query string *Q* from Fig. 1. Note how, for example, color 19 is a false positive for the first of the two reasons described above: it belongs to both $C_{4}(\mu_{1})$ and $C_{4}(\mu_{3})$ and has a score of 6>⌊τ(|Q|−k+1)⌋=4. This means that minimizers CCGG and ACCT appear in $R_{19}$, leading to color 19 to be associated to all *k*-mers having these two minimizers (in the example, $x_{1}$ and $x_{4-8}$). On the other hand, as an example of alien minimizer lookups, we reconsider the example from Fig. 1. There, *k*-mer $x_{9}$ does not appear in any document of R. Let us further assume that this is so because its minimizer, AAGC, is an alien minimizer. While the returned color set $C_{4}(\mu_{4})$ can be any of the indexed color sets, the fingerprint matching strategy correctly identifies it as an alien minimizer. Note how this prevents the color 8 to gain a score of 8.

The expectation is that false positives do not occur in more than τ·100% of the color sets of the *k*-mers of *Q*, and are thus not reported in the final result $\tilde{R}(Q,\tau )$. We explore the mechanics of alien false positives in greater depth in the Appendix (Fig. 5 and Section 1.4), available as supplementary data at *Bioinformatics Advances* online.

## 5 Results

Kaminari is written in C++ and was compiled with gcc 14.2.0 for the experiments reported here. Reproducibility scripts, including those to download all tested collections, to build and query the indexes can be found at github.com/vicLeva/benchmarks_kaminari.

We report the performance of Kaminari in terms of disk size, query time, and resource usage during construction. Effectiveness is measured using the RBO similarity, as stated in Section 2. For all experiments, we considered canonical *k*-mers, with k=31, τ=0.8 for queries. We used a minimizer size of 19. Results varying this parameter are proposed in the Appendix (Fig. 1), available as supplementary data at *Bioinformatics Advances* online.

**Hardware.** All experiments were conducted on a GenOuest platform on a node with 4×8 cores Xeon E5-2660, clocked at 2.20 GHz and with 1.5 TB of memory, running CentOS Linux 7 (Core) server with a 64-bit architecture (kernel version 3.10.0).

**Datasets.** We consider five collections characterized by different file counts, average lengths, and internal similarity. Basic statistics are provided in Table 1.

**Table 1:** Some basic, approximate, statistics for the tested collections for k=31 and m=19 as minimizer length.

	Ecoli	Salmonella	Human	Gut	Sea water	Refseq
**Colors**	3682	10 000	60	10 000	12	25 321
**Distinct *k*-mers ($\times 10^{6}$)**	259	633	5917	7766	25 714	29 177
** Min ($\times 10^{6}$)**	0.05	0.14	2423	0.41	1579	0.43
** Max ($\times 10^{6}$)**	11.01	13.21	2574	4.18	4122	47.85
** Avg ($\times 10^{6}$)**	5.06	4.84	2528	1.92	2478	12.22
**Distinct minimizers ($\times 10^{6}$)**	20	58	347	763	2485	2884
**Distinct color sets ($\times 10^{6}$)**	2.866	2.847	6.640	72.474	0.002	309.995

- Ecoli: 3682 *Escherichia coli* genomes.
- Salmonella: 10 000 *Salmonella enterica* genomes.
- Human: 60 whole human genomes (30 paternal haplotypes; 30 maternal).
- Gut: 10 000 Gut metagenome-assembled genomes (MAG).
- Sea-Water: A collection of 12 metagenomics non-assembled samples from sea water.
- Refseq: A metagenomic collection consisting of 25 321 Archaea and Bacteria genomes in the Refseq database (O’Leary *et al.* 2015).

**Competitors.** We compared the performance of Kaminari against two exact tools, Fulgor and MetaGraph, and three approximate tools [rambo is excluded due to its index size being up to twice that of COBS, and HowDe-SBT is not included as its construction and queries are significantly slower than those of COBS (Bingmann *et al.* 2019)]: COBS, kmindex and Raptor. More details about these tools in the Appendix, available as supplementary data at *Bioinformatics Advances* online.

Among the tested tools, Fulgor and MetaGraph are exact solutions, yielding no false positive matches. They serve as baselines for evaluating the tradeoffs of approximate solutions. Additionally, we compared the exact rankings from Fulgor with those from non-exact tools using the RBO measure to assess the latter’s effectiveness.

All indexes were evaluated using the C++ implementations provided by the authors. Default parameters were employed unless otherwise noted, and tools were provided 32 threads to operate. We report in the Appendix, available as supplementary data at *Bioinformatics Advances* online, the tested tool versions and the exact parameters used.

### 5.1 Efficiency

**Index size.**  Table 2 shows the disk size of each index and raw data. With the exception of MetaGraph, Kaminari consistently produces the smallest index, often an order of magnitude smaller. Results show that Kaminari performs well across various data types, including assembled bacterial and eukaryotic genomes, MAG genomes, and complex raw data. Although MetaGraph generates compact indexes, it suffers from significantly longer query times, as shown in the next section.

**Table 2:** Comparisons of index size on disk and raw data size (compressed with gzip).^a^

	Ecoli	Human	Salmonella	Gut	Sea-water	Refseq
**Raw data size (gzip)**	5.52	48.53	14.17	5.72	38.79	29
**Kaminari**	0.50	1.13	0.83	4.73	4.51	21.45
**Fulgor**	1.35	4.67	2.28	12.23	37.54	48.85
**COBS**	7.01	64.07	17.59	6.95	109.50	36.82
**kmindex**	2.91	11.02	9.49	5.43	4.51	29.10
**Raptor**	2.63	17.75	7.03	4.00	9.04	18.61
**MetaGraph**	0.33	3.09	0.60	3.88	11.05	18.03

a All values are in gigabytes. All tools were used with default parameters.

**Query performance.** The second key result is Kaminari’s query speeds, reported in Table 3 (and Table 1 in the Appendix, available as supplementary data at *Bioinformatics Advances* online). These results show the total elapsed time to perform 50 000 queries for each dataset, each query consisting of a sequence composed of 1000 bases. In case of so-called “*positive queries*” (Table 3), these sequences are randomly taken from the documents used to build the index, with the constraints of being contiguous and made of valid nucleotides. On the other hand, “*negative queries*” (Table 1 in the Appendix, available as supplementary data at *Bioinformatics Advances* online) are random sequences absent from the documents. Results with different query lengths are proposed in the companion repository.

**Table 3:** Time and peak memory usage for 50 000 positive queries (1000 base pairs each), using τ=0.8.^a^

	Ecoli		Human		Salmonella		Gut		Sea-water		Refseq	
	secs	GB	secs	GB	secs	GB	secs	GB	secs	GB	secs	GB
**Kaminari**	7	0.6	1	1.2	21	1.2	1	3.4	2	4.6	6	21.16
**Fulgor**	21	1.5	8	4.7	62	2.4	16	12.3	29	37.6	39	48.9
**COBS**	80	7.4	103	64.1	166	18.6	52	7.9	121	109.5	59	39.2
**kmindex**	88	24.2	19	2.5	328	60.3	91	61.2	11	3.1	219	164.1
**Raptor**	3	2.7	10	17.8	8	7.1	2	4.1	5	9.1	11	18.7
**MetaGraph**	3619	0.5	262	3.1	13 065	0.8	200	4.0	27	11.1	893	15.0

a All tools were used with default parameters.

RAM usage reflects the size of the index for all tools, except for kmindex. This tool, in fact, does not load the full index in memory. Additionally, it is optimized so that parts of the index are mapped to RAM and remain in RAM as long as memory is available, limiting the number of disk accesses. This explains its higher RAM usage with 50 000 queries.

The MetaGraph results indicate prohibitive query times, up to ≈600× slower than Kaminari when performing positive queries. Raptor also shows good time performance, however, its index sizes and, consequently its RAM usage is up to an order of magnitude larger than that of Kaminari.

Overall, Kaminari results are always among the best ones, if not the best. In contrast, all other tested solutions have at least one instance where query time or RAM usage is an order of magnitude greater than that of Kaminari.

**Construction performance.**  Table 2 of the Appendix, available as supplementary data at *Bioinformatics Advances* online, shows that the construction results for Kaminari are good, if not the best, for genomic datasets (Ecoli, Human, Salmonella). For more complex datasets like Gut and Sea-Water, tools such as kmindex perform better due to their specialized construction algorithms.

### 5.2 Effectiveness

In this section, we provide effectiveness results using all tools with their default parameters. We believe this information is essential from a user perspective. However, these comparisons might initially appear unfair as, under these conditions, false positive rates (FPRs) and index sizes are not directly comparable across tools with different internal parameters. This is why we also propose two experiments, presented at the end of the section, in which we fix one of these two parameters at a time.

**False positives evaluation.** To evaluate effectiveness, we treat all methods as black boxes aiming to solve the same fundamental problem: identifying documents that share a specific fraction of *k*-mers with the query. Using a threshold τ=0.8, we define the ground truth via an exact index: a document is a true positive if and only if it explicitly contains at least 80% of the query’s *k*-mers. Consequently, a document is defined as a false positive if a tool reports it as a match, whereas the exact index confirms it contains fewer than 80% of the *k*-mers. This definition allows for a uniform comparison based on the end result for the user—the percentage of shared *k*-mers—regardless of whether the underlying method uses minimizers, Bloom filters, or other heuristics.

Table 4 shows that Kaminari and COBS produce similar results, though their false positives arise from distinct sources (minimizer collisions vs. Bloom filter saturation). In contrast, Raptor accumulates imprecision from both, leading to higher false positive counts. The Findere (Robidou and Peterlongo 2021) method allows kmindex to attain the best precision, *albeit* with significantly slower query times. For negative (random) queries, we observed that all tested tools reported 0 false positives.

**Table 4:** False positive rates (%), using default parameters for all tools (recalling that index sizes for COBS, kmindex, and Raptor are ≈1 to 21× bigger than for Kaminari).

	Ecoli	Human	Salmonella	Gut	Refseq
**Kaminari**	25.61	1.24	32.08	5.83	14.53
**COBS**	25.83	0.87	31.64	5.84	14.12
**kmindex**	5.81	1.06	5.00	0.60	1.55
**Raptor**	31.14	1.27	36.67	7.97	16.68

**RBO results.** False positives arise from over-estimations that cause a document to incorrectly exceed the threshold τ. We argue that the raw count of false positives can be misleading, and that the real impact on the user is better captured by the Rank Biased Overlap (RBO) metric.

We compared Kaminari answers to the ground truths provided by Fulgor by using the RBO metric to estimate the impact of false positives in applications where top hits are the most informative. For every query, we computed $\text{RBO}(R(Q,\tau ),\tilde{R}(Q,\tau ),p)$, with R(Q,τ) being the ordered list of documents generated by the exact tool (Fulgor) and $\tilde{R}(Q,\tau )$ the one generated by approximate tools, including Kaminari. The RBO value depends on a parameter *p*. We explain in the Appendix, available as supplementary data at *Bioinformatics Advances* online how this parameter was determined.

While RBO can be applied to very short lists (e.g. size <10), it is not very informative in such cases due to limited overlap measurement opportunities. Therefore, we present RBO metrics in Table 5 only for lists of size ≥10. We did not include Sea-Water results as the dataset contains only 12 samples. Similarly, Raptor is excluded due to its lack of ranked output, rendering RBO evaluation infeasible.

**Table 5:** RBO values distribution for positive queries, for truth lists of size ≥10.^a^

	RBOs	≤0.50	[0.50;0.95]	[0.95;1.00]
	Kaminari	0	1	99
**Ecoli**	COBS	1	19	80
	kmindex	0	14	86
	Kaminari	11	15	74
**Human**	COBS	0	4	96
	kmindex	7	12	81
	Kaminari	0	1	99
**Salmonella**	COBS	5	6	89
	kmindex	2	2	96
	Kaminari	1	7	92
**Gut**	COBS	0	29	71
	kmindex	2	6	92
	Kaminari	0	13	87
**Refseq**	COBS	8	41	51
	kmindex	1	16	83

a In each column, we report the proportion (%) of queries whose RBO value are in the specified range. Full results are provided in the Appendix (Fig. 2), available as supplementary data at *Bioinformatics Advances* online.

All datasets and tools show a significant skew toward high RBO values, with most queries clustering near an RBO of 1. This suggests that tools addressing Problem 2 produce rankings closely resembling the ground truth. Except for the Human dataset, Kaminari consistently achieves the highest RBO values across biological domains, demonstrating its robustness.

In the specific case of the Human dataset, the RBO metric encounters limitations due to the extreme similarity of samples, leading to subtle variations in query answers where minor differences can alter rankings. Nevertheless, as shown previously, the false positive rate for this dataset remains low at 1.24% for Kaminari, thus preserving the biological significance of the findings despite these ranking fluctuations. To validate this claim, we performed a test using a minimizer length m=23 instead of m=19, providing a higher resolution. As expected, the ranking quality improved. The proportion of queries in the highest accuracy bin rose from 74% to 82%. Although the index size increased from 1.2 GB to 1.8 GB, it remains order(s) of magnitude smaller than competing indexes for the same dataset, such as COBS (11 GB) and kmindex (64 GB).

#### 5.2.1 Effectiveness fixing the index sizes

In this section, for all tested tools, we fixed the index size to the one obtained by Kaminari. In this configuration, false positive rates, shown in Table 6, highlight that on genomic data, for the same disk size budget, Kaminari generates the least amount of false positives. On the metagenomic Gut dataset, kmindex performs better despite being ≈9× slower to query. RBO results (Fig. 3 in the Appendix, available as supplementary data at *Bioinformatics Advances* online) additionally show that in this setup, Kaminari provides more precise ranking of the results, for any of the considered dataset.

**Table 6:** False positive rates (%), using the same index size for all tested tools.

	Ecoli	Human	Salmonella	Gut	Refseq
**Kaminari**	25.61	1.24	32.08	5.83	14.53
**COBS**	76.67	1.92	59.27	10.57	20.93
**kmindex**	76.59	1.92	59.21	0.79	2.53
**Raptor**	62.91	1.92	58.11	7.99	16.41

#### 5.2.2 Effectiveness fixing the FPR

In Table 7, we adjusted the parameters of the approximate tools in order to achieve 10% of false positives in queries. Human and Sea-Water datasets were not included because 10% could not be reached due to the data redundancy and documents number. Results are similar to Table 2 in the sense that Kaminari provides the best results, except for metagenomes for which it nevertheless remains competitive.

**Table 7:** Index size (GB), while empirically producing results with 10% of false positives.^a^

	Ecoli	Salmonella	Gut
**Kaminari**	0.94	1.73	3.44
**COBS**	20.29	65.28	4.52
**kmindex**	2.20	6.51	2.71
**Raptor**	15.76	NA	2.93

a With k=31, Raptor could not reach 10% of false positives for Salmonella. More details about the parameters used can be found in the Appendix, available as supplementary data at *Bioinformatics Advances* online.

## 6 Conclusions and future work

In this work, we introduced Kaminari, a novel approximate approach for indexing sets of genomic sequences. By leveraging the properties of *k*-mer minimizers, Kaminari achieves significant improvements over traditional Bloom filter-based solutions in terms of both memory efficiency and query performance. We believe this approach will enable the creation of indexes for massive datasets, in the terabyte regime, while significantly reducing query time.

Some tools are more suited to indexing and querying a set of closely related genomes [e.g. Fulgor (Fan *et al.* 2024)] while others are better tailored for complex non-assembled datasets [e.g. kmindex (Lemane *et al.* 2024)]. On genomic datasets Kaminari always generates the smallest index [with the notable exception of MetaGraph (Karasikov *et al.* 2020, 2022) which, however, suffers from very slow query times]. On metagenomic datasets, kmindex achieves the best FPR for fixed index size, despite being approximately 9× slower to query. Overall, Kaminari consistently ranks as one of the fastest tools across all data types, generating the smallest indexes (or the lowest FPR), often achieving the top performance and providing qualitative rankings of results. This robustness represents a key advantage when indexing heterogeneous and/or poorly characterized datasets.

This work pioneers the use of Rank-Biased Overlap (RBO) metric to evaluate similarity between ranked lists of results in the context of genomic sequences. Unlike traditional false positive measurements, RBO quantifies how approximation impacts result ordering—a crucial metric for assessing bias in non-exact indexing methods. We propose this evaluation framework as a new standard for measuring approximate indexing quality.

While Kaminari may generate some false-positive answers, RBO results showed that the impact on the ranking of colors is small. For instance, results on Ecoli show that 99% of queries present a RBO higher than 0.9, indicating the reliability of the top results.

Future work will study the use of partitioned indexes in external-memory for scaling up to even larger collections, and *repetition-aware compression* (Campanelli *et al.* 2024) to compress the color sets even further.

## Supplementary Material

vbag120_Supplementary_Data

## Data Availability

Reproducibility scripts, including sources to download all tested collections and to build and query the indexes can be found at github.com/vicLeva/benchmarks_kaminari. All tools, their version and documentation can also be found in the companion repository.
