# Supplementary material for: Kaminari: a frugal colored index for approximate k-mer queries
Source: Bioinform Adv. 2026 Apr 26;6(1):vbag120. doi: 10.1093/bioadv/vbag120 (PMC13275129; doi:10.1093/bioadv/vbag120)
Supplement: vbag120_Supplementary_Data [file vbag120_supplementary_data.pdf]

1. Appendix

1.1. Rank-biased overlap (RBO)

Given two ranked lists of infinite length, A and B, Webber et al. [Webber et al., 2010] define their *rank-biased overlap* (RBO, henceforth) as a measure of their similarity. Let $X_d = |A[1..d] \cap B[1..d]|$ be the overlap between the prefixes of length d. For a given parameter $0 < p < 1$, the similarity is defined as

$$\text{RBO}(A, B, p) = \frac{1-p}{p} \sum_{d=1}^{\infty} \frac{X_d}{d} p^d$$

where X_d/d is a measure of "agreement" between the prefixes of length d. Clearly, the similarity lies in $[0, 1]$: a value of 0 means that the two rankings are disjoint and 1 means that they are identical.

Bounding RBO. Although RBO is defined over infinite-length ranked lists, the summation must be truncated at a given depth D in practice. Call RBO@D (read "RBO at depth D") the truncated RBO value. It is easy to see that RBO@D provides a lower bound to the true value of RBO, i.e., RBO > RBO@D, if RBO@$D > 0$. However, Webber et al. [Webber et al., 2010] derive a tighter lower bound as

$$\text{RBO}_{\text{MIN}}(A, B, p, D) = \frac{1-p}{p}\Big(\sum_{d=1}^{D} \frac{X_d - X_D}{d} p^d - X_D \ln(1-p)\Big).$$

In this paper, we use the above formula with the largest possible D, that is $D = \min\{|A|, |B|\}$. We set $A = R(Q, \tau)$ and $B = \widetilde{R}(Q, \tau)$. Since $\widetilde{R}(Q, \tau) \supseteq R(Q, \tau)$, we have $D = |A|$.

Determining p for RBO computations. The choice of p is of utmost importance for RBO as it influences the result. Webber et al. [Webber et al., 2010] derived a formula to retrieve the weight of a prefix of the lists according to the bias parameter p:

$$W_{\text{RBO}}(p, d) = 1 - p^{d-1} + \frac{1-p}{p} \cdot d \cdot \left(\ln\left(\frac{1}{1-p}\right) - \sum_{i=1}^{d-1} \frac{p^i}{i} \right).$$

As an example, $W_{\text{RBO}}(0.85, 17) = 0.9846$ means that the first 17 elements of the lists will weight for 98.46% of the RBO value. For positive queries, the length of the lists can vary from 1 to N. With such a variability, we made the choice to adapt p according to the length of the lists. More precisely, for every query, we fixed p so that $W_{\text{RBO}}(p, \lceil 0.1 \cdot |R(Q, \tau)| \rceil) \approx 0.9$. In other words, we want the top 10% of the list's elements to explain 90% of the RBO value. To determine p for a given d so that $W_{\text{RBO}}(p, d) \approx 0.9$, we can exploit the following fact.

Fact 1 For fixed d, $W_{\text{RBO}}(p, d)$ is decreasing as p increases.

Proof. We show that $\frac{d}{dp}(W_{\text{RBO}}(p, d)) < 0$. For the first term $1 - p^{d-1}$, we have $\frac{d}{dp}(1 - p^{d-1}) = -(d-1)p^{d-2}$. Call $f(p, d) = \ln\left(\frac{1}{1-p}\right) - \sum_{i=1}^{d-1} \frac{p^i}{i}$ and consider the second term $\frac{1-p}{p} \cdot d \cdot f(p, d)$. We have $\frac{d}{dp}\left(d \cdot f(p, d) \cdot \frac{1-p}{p}\right) = d\left(f'(p, d) \cdot \frac{1-p}{p} - f(p, d) \cdot \frac{1}{p^2}\right)$. Now, we compute $f'(p, d) = \frac{d}{dp}\left(\ln\left(\frac{1}{1-p}\right)\right) - \frac{d}{dp}\sum_{i=1}^{d-1} \frac{p^i}{i} = \frac{1}{1-p} - \sum_{i=1}^{d-1} \frac{d}{dp}\left(\frac{p^i}{i}\right) = \frac{1}{1-p} - \sum_{i=1}^{d-1} p^{i-1} = \frac{1}{1-p} - \frac{1-p^{d-1}}{1-p} = \frac{p^{d-1}}{1-p}$. Hence by simplifying, we obtain that $\frac{d}{dp}(W_{\text{RBO}}(p, d)) = p^{d-2} - \frac{d}{p^2} \cdot f(p, d)$.

To conclude, we show that $\frac{d}{dp}(W_{\text{RBO}}(p, d)) < 0 \iff \frac{d}{p^2} \cdot f(p, d) > p^{d-2} \iff f(p, d) > \frac{p^d}{d}$. Recall that, for $0 < p < 1$, the Taylor expansion of the logarithm is $\sum_{i=1}^{\infty} \frac{p^i}{i} = \ln\left(\frac{1}{1-p}\right)$. We can rewrite $\sum_{i=1}^{\infty} \frac{p^i}{i}$ as $\sum_{i=1}^{d-1} \frac{p^i}{i} + \frac{p^d}{d} + \sum_{i=d+1}^{\infty} \frac{p^i}{i} = \ln\left(\frac{1}{1-p}\right) \iff f(p, d) = \ln\left(\frac{1}{1-p}\right) - \sum_{i=1}^{d-1} \frac{p^i}{i} = \frac{p^d}{d} + \sum_{i=d+1}^{\infty} \frac{p^i}{i}$. The latter quantity is clearly larger than $\frac{p^d}{d}$ as $\sum_{i=d+1}^{\infty} \frac{p^i}{i} > 0$. □

Thus, we simply calculate the function $W_{\text{RBO}}(p, d)$ for increasing $p \in (0, 1)$ and return the first value of p such that $W_{\text{RBO}}(p, d) \approx 0.9$. In practice, we consider the values $W_{\text{RBO}}(i \cdot \varepsilon, d)$ for $i = 1, \ldots, \lceil (1-\varepsilon)/\varepsilon \rceil + 1$ and return the first (i.e., largest) value $p = i \cdot \varepsilon$ for which $W_{\text{RBO}}(p, d) < 0.9$. The smaller ε, the better the approximation.

Interpretation and effectiveness. The parameter p affects the "importance" given by the top-ranked elements and has a natural probabilistic interpretation. In fact, p can be regarded as the probability that a user considers the next element in the ranking: a low p value indicates that the user is satisfied with the top results only; vice versa, a high value indicates the user's willingness to consider more elements down in the ranking.

The RBO measure is particularly useful in scenarios where the *order* of the elements in the lists matters *more than their presence* lower down the ranking. For example, $\widetilde{R}(Q, \tau)$ could contain a large amount of false positives but appearing at low rank positions, while the top-ranked colors could indeed be identical to those in $R(Q, \tau)$. A high RBO value thus indicates that — even in the presence of false positive matches — the ranking produced by the proposed index aligns closely with the true ranking. This ensures that the most relevant documents still appear at the top, preserving the overall utility of the retrieval process despite approximation errors.

Example. Consider the ranked list $R = [4, 14, 13, 22, 3, 5, 9, 2]$ from Figure 1 (main text) and $\widetilde{R} = [4, 5, 13, 14, 22, 8, 19, 2, 3, 9, 18]$ from Figure 3 (main text). We have $D = \min(|R|, |\widetilde{R}|) = |R| = 8$. Using $p = 0.5$, we have an RBO similarity of 0.801739 and RBO_{MIN} is 0.804121. With a lower p, for example $p = 0.3$, the two scores are higher and more similar to each other: 0.867608 and 0.867650, respectively.

1.2. Tool versions and used parameters

- Kaminari: commit `305f2c5`, parameters: `-m 19`
- Fulgor: commit `5ac5699`, parameters: `-m 19`.
- COBS: commit `2fbb044`, parameters: `--compact-construct`.
- kmindex: version `0.5.2`, parameters: `-k 25`, `-z 6`. To query 31-mers, kmindex considers 25-mers using the findere approach [Robidou and Peterlongo, 2021].
- Raptor: commit `e7b5a35`, parameters: `--kmer 19 --window 31`
- MetaGraph: version `0.3.6` (commit `5c2a12b`). In particular, we built the indexes following the methodology from [Fan et al., 2024] (reproducible with the workflow available at `https://github.com/theJasonFan/metagraph-workflows`): the indexes use the "relaxed row-diff" BRWT data structure [Karasikov et al., 2020], which is the most compact variant of MetaGraph.

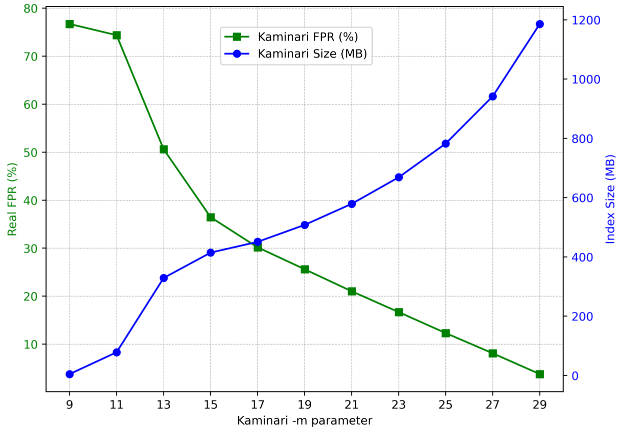

Fig. 1. Kaminari index size and FPR measured on positive queries by varying the m parameter when built on the Ecoli dataset. k is fixed to 31.

About Kaminari, the choice of m impacts the size and the false positive rate of the index. Figure 1 shows the trade-off between performance and precision. We think $m = 19$ is a reasonable choice considering our needs.

1.3. Additional results

1.3.1. Performances for negative queries

Similar conclusions apply to those drawn in the main text to negative queries (Table 1), with two notable differences. Firstly, Fulgor excels in quickly detecting the absence of queried k-mers due to the SSHash data structure [Pibiri, 2022]. Secondly, MetaGraph queries do not experience the significant computation time issues seen with positive queries.

1.3.2. Time and RAM for building indexes

Table 2 provides index construction times and peak memory usage across tools and datasets.

1.3.3. RBO distribution, full results

Figure 2 shows RBO results distribution, when using default parameters of tested tools. This is an extension of results presented in Table 5 in the main text.

1.3.4. RBO distribution, using equal index sizes for all tools

Figure 3 shows RBO distribution while using the same index size for all tested tools. One result stands out: the COBS result for Human dataset, this can be explained by the extreme value of 0.999999999 for the `--false-positive-rate` parameter used to reach the target index size. Although other results in Figure 3 are meaningful, this one can be sidelined.

1.3.5. Raptor's trade-off

Despite proposing ranked results, Raptor appears to be a serious competitor when it comes to performances (index size, query speed). In fact, it can reach Kaminari's index size under certain parameters. Although as shown in Figure 4, when Raptor has the same index size for Ecoli than Kaminari (blue curves), its false positive rate is almost twice as big (green curves). In fact, with any parameters, Kaminari acts like a lower bound for both size and FPR for Raptor.

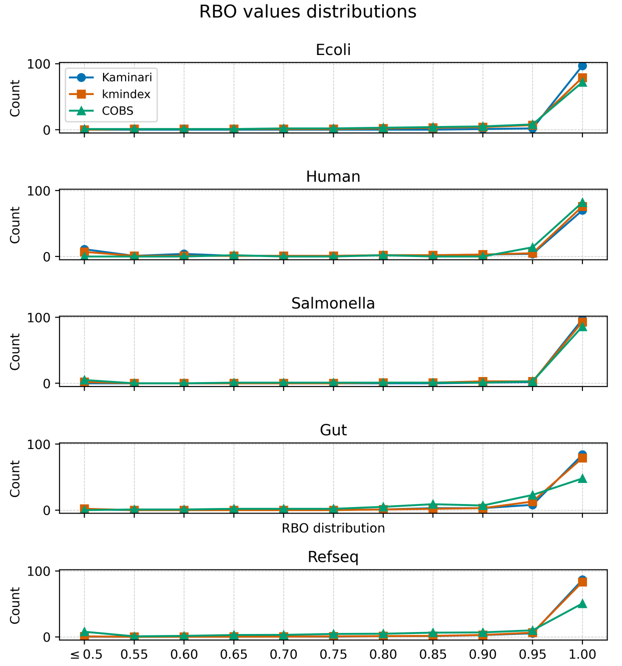

Fig. 2. RBO values distribution for positive queries, for truth lists of size ≥ 10. Each point shows the sum of the percentage of queries from its x value (included) to the previous one (excluded). The leftmost point sums the percentage of queries whose RBO values are in $[0, 0.5]$.

1.3.6. Parameters

Table 6 and Table 7 from main text present results where we tweaked some parameters to reach certain results. In any case, we kept $k = 31$. For COBS and kmindex, we changed the parameters `--false-positive-rate` and `--bloom-size`, respectively, for both experiments. About Raptor, in Table 6, we kept $m = 19$ (called `--kmer-size` in Raptor) as it corresponds to Kaminari's m value, then we modified the `--false-positive-rate` parameter. In Table 7, as we modified m in Kaminari, we also did in Raptor. Thus, in this second experiment, we tweaked `--kmer-size` and `--false-positive-rate` for Raptor. Note that for Salmonella, even with `--kmer-size 30` and `--false-positive-rate 0.0001`, a FPR of 10% could not be reached. Exact parameters are summarized in the companion repository. We excluded Sea-Water for Table 6 and 7 and Human for Table 7 because the small number of documents is not reliable to compute statistics for false positive rate, and the Human dataset is too redundant to reach 10% of false positives.

1.4. Extended Analysis of False Positive Sources

In the main text, we identified two distinct sources of false positives arising from the index structure. Here, we elaborate on their specific behaviors and how they affect query scoring differently depending on the nature of the query (positive vs. negative).

1.4.1. Characterization of Error Types

The two types of false positives differ fundamentally in their origin:

	Ecoli		Human		Salmonella		Gut		Sea-Water		Refseq	
	seconds	GB	seconds	GB	seconds	GB	seconds	GB	seconds	GB	seconds	GB
Kaminari	1	0.6	1	1.2	1	0.9	1	4.7	2	4.6	4	17.7
Fulgor	1	1.5	8	4.7	2	2.3	13	12.3	26	37.6	22	48.9
COBS	56	7.4	98	64.1	55	18.6	52	7.9	118	109.5	49	39.2
kmindex	41	24.2	17	2.5	110	60.3	84	61.2	11	3.0	231	164.8
Raptor	1	2.7	7	17.8	5	7.1	2	4.1	4	9.1	9	18.7
MetaGraph	7	0.4	18	3.2	11	0.7	23	4.0	35	11.1	29	14.9

Table 1. Total elapsed time (seconds) and peak memory usage (GB) for 50,000 negative queries (1000 base pairs), using $\tau = 0.8$. See Table 2 (main text) for color code.

	Ecoli		Human		Salmonella		Gut		Sea-Water		Refseq	
	h:mm:ss	GB	h:mm:ss	GB	h:mm:ss	GB	h:mm:ss	GB	h:mm:ss	GB	h:mm:ss	GB
Kaminari	0:01:54	8.05	0:24:03	17.61	0:05:02	8.05	0:29:13	51.46	0:27:54	128.63	2:04:05	232.55
Fulgor	0:08:36	16.45	0:38:44	219.10	0:14:45	21.84	0:53:04	50.17	2:17:00	153.70	3:42:45	156.51
COBS	0:02:19	6.09	1:02:43	64.07	0:26:27	35.21	0:04:05	6.38	1:13:36	27.38	0:15:59	28.36
kmindex	0:07:07	3.71	0:37:01	8.12	0:38:36	3.38	0:19:29	10.24	0:08:48	43.74	0:53:34	27.07
Raptor	0:01:03	4.07	1:23:36	22.54	0:04:55	8.50	0:02:53	7.55	0:35:21	18.04	0:15:27	28.67
MetaGraph	0:44:50	141.65	3:34:21	284.46	2:39:49	257.08	2:17:54	148.04	2:36:16	256.18	1:27:36	259.18

Table 2. Index construction time (h:mm:ss) and peak memory usage (GB). See Table 2 from the main text for color code.

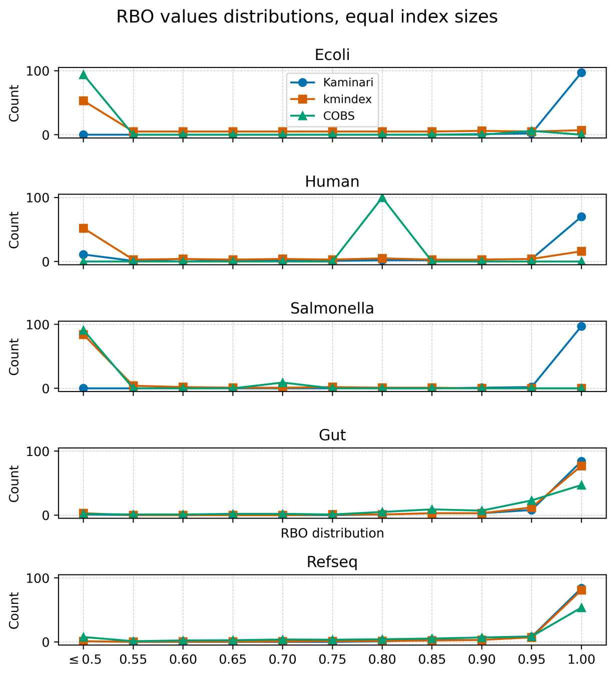

Fig. 3. RBO values distribution for positive queries, for truth lists of size ≥ 10. In this setup index sizes are identical and are equal to Kaminari's one. See Figure 2 for details on this result representation.

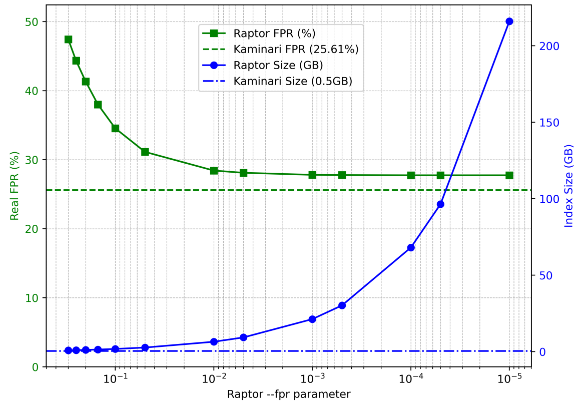

Fig. 4. Raptor index size and FPR measured on positive queries by varying the FPR parameter when built on the Ecoli dataset. Kaminari index size and FPR are parameter independent and are indicated by dashed lines.

- **Type 1 (Shared Minimizers):** This error is deterministic and driven by sequence similarity. It occurs when the query k-mer x is distinct from an indexed k-mer y, but they share the same minimizer $\text{Minimizer}(x) = \text{Minimizer}(y)$. In this scenario, the retrieved color set $C_m(\text{Minimizer}(x))$ is a valid set existing in the biological data. This type of FP is predominant in positive or related queries, where the query k-mers generate minimizers that exist in the index.
- **Type 2 (Alien Minimizers):** This error is probabilistic and driven by the properties of the Minimal Perfect Hash Function (MPHF). Since an MPHF maps the entire universe of possible minimizers to the range $[1, N]$ without keeping track of the original key set, a non-existent ("alien") minimizer μ is inevitably mapped to a valid slot $f(\mu)$. The retrieved color set is essentially a random selection from the index. This type of FP is predominant in negative queries (random or unrelated sequences), where the generated minimizers are unlikely to exist in the reference.

1.4.2. *Accumulation of false positives and filtering via fingerprinting*

While a single Type 2 error yields a random color set, a full query consists of many k-mers. In a negative query, every k-mer generates an alien minimizer, resulting in a sequence of random color set retrievals.

If the threshold τ is low (e.g., $\tau = 0.1$) and the bit-fingerprint b is not utilized, these random "votes" can accumulate in specific document scores simply by chance, especially in indexes with low diversity where large color sets are common. This results in the tool reporting a match for a completely unrelated sequence.

However, the introduction of the b-bit fingerprint specifically targets and filters out these Type 2 errors. By comparing some extra bits of the hash signature, we can distinguish between a valid index slot and an MPHF collision, effectively filtering out alien minimizers before they contribute to document scores.

1.4.3. *Experimental validation*

To empirically demonstrate the impact of Type 2 errors and the efficacy of the b-bit filter, we performed an experiment using the Human dataset. Due to its lower color set diversity compared to metagenomes, this dataset represents a "worst-case" scenario for the accumulation of false positives from alien minimizers. We queried this index with 50,000 random negative sequences.

Figure 5 illustrates the combined effect of the query threshold τ and the fingerprint size b on these errors. We observe that for a standard threshold (e.g., $\tau = 0.8$), increasing b by just a single bit is sufficient to eliminate all remaining false positives.

It is important to note that the Human dataset is an outlier in this regard: in our experiments, no false positives were observed for any other dataset at $\tau = 0.8$, even with $b = 0$. This confirms that the Human index possesses a specific redundancy that makes it more prone to so-called alien false positives. However, even in this extreme case, false positives can be neutralized at a negligible spatial cost. Since the index contains approximately 347 million minimizers, increasing b by 1 adds only $\approx$ 41 MB to the index size (3.7% increase over the baseline 1114 MB).

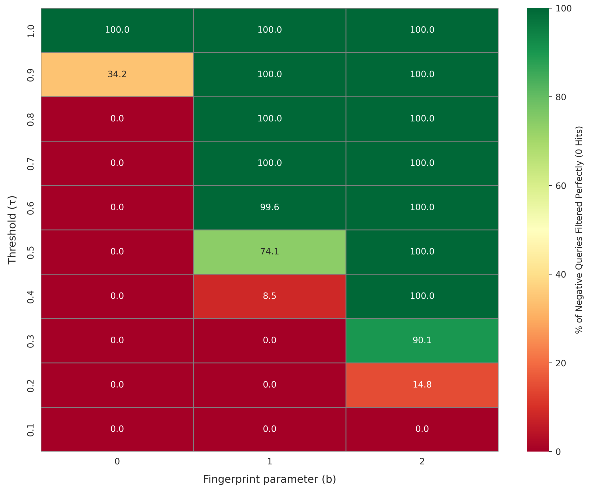

Fig. 5. Heatmap showing the percentage of results that correctly returned zero dataset identifier (an empty result set $R(Q, \tau)$) for negative queries (random sequences) on the human dataset. The x-axis represents fingerprint size b (in bits) used during construction. The y-axis represents the threshold τ used at query. A value of 100 indicates perfect filtration (all 50,000 queries returned no matches). For example, the value 74.1 at $b = 1$ and $\tau = 0.5$ means that 74.1% of the negative queries were successfully filtered out, while the remaining 25.9% yielded at least one false positive document.

References

J. Fan, J. Khan, N. P. Singh, G. E. Pibiri, and R. Patro. Fulgor: A fast and compact k-mer index for large-scale matching and color queries. *Algorithms for Molecular Biology*, 19(1):1–21, 2024.

M. Karasikov, H. Mustafa, A. Joudaki, S. Javadzadeh-no, G. Rätsch, and A. Kahles. Sparse Binary Relation Representations for Genome Graph Annotation. *Journal of Computational Biology*, 27(4):626–639, Apr. 2020.

G. E. Pibiri. Sparse and skew hashing of k-mers. *Bioinformatics*, 38(Supplement_1):i185–i194, 06 2022. ISSN 1367-4803.

L. Robidou and P. Peterlongo. findere: Fast and precise approximate membership query. In *String Processing and Information Retrieval*, pages 151–163, Cham, 2021. Springer International Publishing. ISBN 978-3-030-86692-1.

W. Webber, A. Moffat, and J. Zobel. A similarity measure for indefinite rankings. *ACM Transactions on Information Systems (TOIS)*, 28(4):1–38, 2010.
